# Trichinosis

**Published:** 1912-08

**Authors:** 


Trichinosis, Dr. Geo. H. Runckel, McCloud, Calif., *Calif. state Jour.* of Med., July, 1912. 58 cases due to eating salame sausage. 1 death. Eosinophilia 10—40%, usual symptoms, numerous active free larvae demonstrated in gastrocnemius of two patients, no adult trichinae found in faeces, sausages demonstrated to be badly infected. Treatment: castor oil, followed by glycerine, 15 cc. every hour; acetozone; quinine; Post-febrile treatment: iron, quinine and strychnine, bismuth, morphine and diarrhoea mixtures p. r. n. Another case of trichinosis in a pregnant woman, apparently cured, had what seemed to be a recurrence following delivery.

